# An integrative view on the systematic position of the cupressophyte *Cephalotaxus*

**DOI:** 10.1002/ece3.10273

**Published:** 2023-07-05

**Authors:** Yong Yang, Zhi Yang, David Kay Ferguson, Jia‐Yi Shong

**Affiliations:** ^1^ Co‐Innovation Center for Sustainable Forestry in Southern China, College of Biology and the Environment Nanjing Forestry University Nanjing China; ^2^ Department of Paleontology University of Vienna Vienna Austria

**Keywords:** Cephalotaxaceae, gymnosperms, integrative taxonomy, phylogenomics

## Abstract

We made an in‐depth review of historical studies of the cupressophyte conifer genus *Cephalotaxus* Siebold & Zucc. with an emphasis on its systematic position. We suggest that the systematic position of the genus is better understood using an integrative approach, so the evolution of phenetic characters is discussed within the context of recent phylogenomics. We propose that the genus should be classified as a separate family Cephalotaxaceae belonging to the clade consisting of Cupressaceae, Cephalotaxaceae, and Taxaceae; the family Cephalotaxaceae is sister to the Taxaceae but not nested within the Taxaceae and is characterized by a unique set of characters including morphology, anatomy, embryology, and chemistry. The family Cephalotaxaceae shows transitional characters between the Cupressaceae and the Taxaceae; the family possesses female cones with a primary cone axis bearing 5–8 pairs of decussate bracts, which is similar to the typical female cones of the Cupressaceae, on the one hand, and may have given rise to the reduced female cone of the Taxaceae with one terminal ovule partially or completely enclosed in a fleshy aril. In parallel, the compound male cone of the Cephalotaxaceae evolved into the seemingly “simple” male cones of the Taxaceae by means of reduction, elimination, and fusion.

## Graphical abstract

Systematic position of the cupressophyte genus *Cephalotaxus* has not been resolved; some contended that it should be classified in Cephalotaxaceae while others suggested that it should be included in Taxaceae. Here, we integrated multiple disciplinary evidence including morphology, development, anatomy, embryology, chemistry, and phylogeny, and concluded that the genus should be classified in a separate family Cephalotaxaceae. We also clarified that the fleshy part of seed in *Cephalotaxus* is actually derived from the thickening and differentiation of the integument as that in cycads and Ginkgo, but not aril homologous to the Taxaceae.

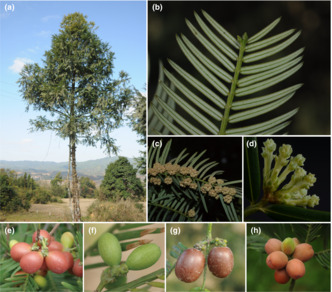

## 1 INTRODUCTION

**FIGURE 1:**
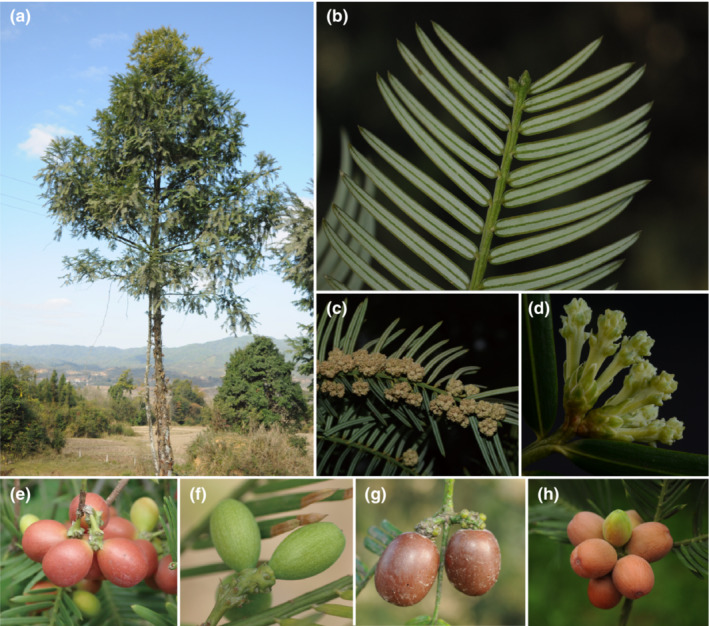
Morphology of Cephalotaxaceae. (a) tree of *Cephalotaxus mannii* displaying the habit, photo by Bing Liu; (b) leafy branch portion of *C. mannii* displaying, photo by Bing Liu; (c) male branch portion of *C. fortunei*, photo by Zhi Yang; (d) female branch of *C. fortunei* showing the clustered young female spikes, photo by Zhi Yang; (e) female cones of *C. sinensis*, photo by Yong Yang; (f) female cone of *C. fortunei*, photo by Yong Yang; (g) female cone of *C. mannii*, photo by Bing Liu; (h) female cone of *C. harringtonia*, photo by Bing Liu.

The conifer genus *Cephalotaxus* Siebold & Zucc. consists of dioecious shrubs or slender trees (Figure 1a, Fu et al., 1999; Page, 1990). The evergreen leaves are linear with nearly parallel sides or tapering gradually toward the tip, entire, opposite or subopposite, arranged in two pectinate ranks due to twisting of petioles, dorsiventrally flattened, upper surface green, lower surface having two pale green or white stomatal bands (Figure 1b), and a conspicuous midvein with a single, large, ventral resin canal (Eckenwalder, 2009; Page, 1990). Male cones are tightly clustered in capitulae in the axils of leaves along both sides of ordinary foliage shoots (Figure 1c), hence the scientific name. The male cone possesses 7–12 spirally arranged microsporophylls each of which bears 2–3 or more egg‐shaped, pendulous microsporangia (Eckenwalder, 2009; Page, 1990). Female organs are pedunculate single or in groups of 2–8 on separate scaly peduncles in axils of bracts (Figure 1d). The female cone consists of a fleshy cone axis with 3–8 pairs of decussate bracts each having two axillary ovules except for the proximal pair. The ovule secretes a pollination drop containing proteins that are important in defense of nutrient‐rich pollination drops from microbial pathogens, promotion and support of pollen tube growth, polysaccharide metabolism during drop production and response to stress (Pirone‐Davies et al., 2016). Mature seeds are pendulous, large, oval or ellipsoid, colorful, fleshy, and drupe‐like (Figure 1e–h, Eckenwalder, 2009; Page, 1990). As in cycads and *Ginkgo*, the seed coat of the Cephalotaxaceae is differentiated into three layers, that is, the inner membranous endotesta, the middle hard sclerotesta, and the outer fleshy sarcotesta (Singh, 1961; Yang et al., 2011).

The plum yew genus contains 7–10 extant species that are restricted to subtropical Asia including East Asia and northern Indo‐Chinese peninsula (Farjon, 2017; Fu, 1984; Fu et al., 1999; Tripp, 1995; Wang et al., 2022). The genus has a long evolutionary history, fossils ascribed to the genus already occurred in the Early Cretaceous, and fossil species of *Cephalotaxus* were once widely distributed throughout the Northern Hemisphere in the Cenozoic (Manchester et al., 2009; Shi et al., 2010; Zhang et al., 2019). The modern restricted distribution of the genus is thus clearly relictual.

Systematic relationships of *Cephalotaxus* Siebold & Zucc. within conifers have been controversial. Some authors included the genus in the Taxaceae (e.g., Christenhusz et al., 2011); some treated it as a separate family, viz. Cephalotaxaceae, either in the suborder Taxineae (Keng, 1975), in the order Taxales (Pulle, 1937), or in the order Coniferae/Coniferales (Pilger, 1926; Takhtajan, 1953), or in the order Cupressales (Yang et al., 2022), or as a separate order Cephalotaxales (e.g., Cheng & Fu, 1978). Multiple disciplinary studies were conducted in the last century, but no comprehensive study of the genus has been undertaken. This study is aimed to assess the historical studies of *Cephalotaxus* and make an integrative study to better understand the systematic position.

## 2 MORPHOLOGY AND DEVELOPMENT

Traditionally, conifers were classified into two major groups: (1) one possessing typical female cones that are normally woody but not fleshy and consisting of a primary cone axis with a variable number of seed scale complexes spirally or decussately arranged, for example, Pinaceae, Araucariaceae, Sciadopityaceae, Cupressaceae; (2) another having atypical female cones that are usually fleshy and reduced with oligomerous seed scale complexes, that is, Podocarpaceae, Cephalotaxaceae, and Taxaceae. In the context of phylogenomic results (Liu et al., 2022; Ran et al., 2018; Stull et al., 2021), the reduced and atypical female cones of conifers were independently derived as an adaptation to zoochorous dispersal, in Podocarpaceae, Cupressaceae, Cephalotaxaceae, and Taxaceae, respectively (Contreras et al., 2017). The structure of the female cone of Cephalotaxaceae does show similarity to some primitive lineages of Cupressaceae, especially the early development of the female cone of *Taiwania cryptomerioides* Hayata: two orthotropous ovules are axillary to a fertile bract (Farjon & Ortiz Garcia, 2003). A very high diversity of cupressoid macrofossils was found in the early Mesozoic (Taylor et al., 2008), so the Cephalotaxaceae may have been rooted in one of them. The Taxaceae are conifers with extremely reduced female reproductive organs that have persisted since the Jurassic (Dong et al., 2020, 2022). How the very simple uniovulate organ originated has been a point of discussion in conifer evolution (e.g., Sahni, 1920; Florin, 1948; Keng, 1975; Shi & Wang, 1989) and remains a mystery. The Cephalotaxaceae are definitely pivotal in understanding the origin and evolution of the reduced female organs of Taxaceae.

**FIGURE 2:**
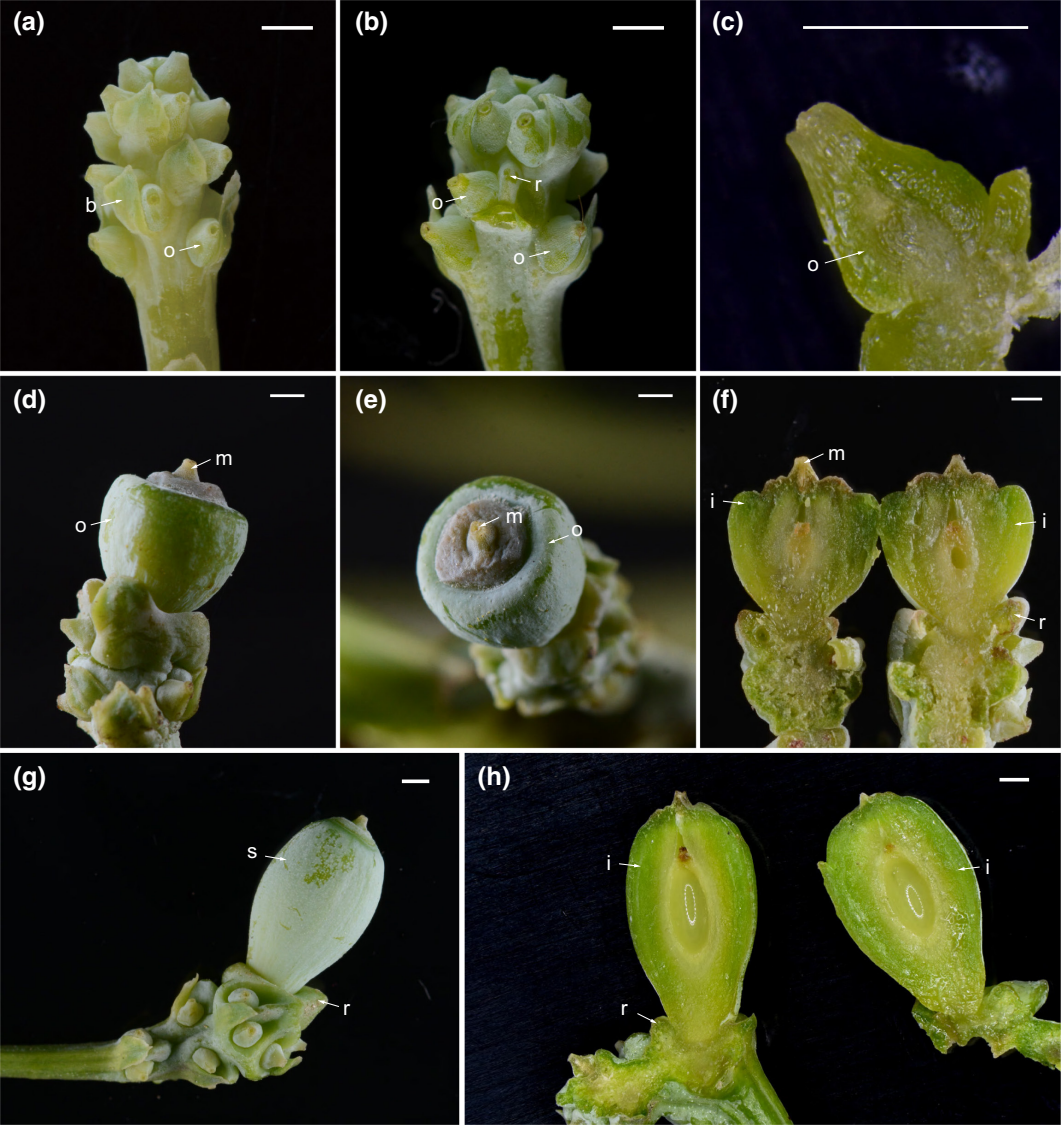
Morphology, structure, and development of female cones of *Cephalotaxus fortunei*. (a) a female cone at the early developmental stage displaying the organization of the bract and axillary orthotropous ovules; (b) a female cone at the early developmental stage with the removal of the bract displaying the ridge between the two axillary orthotropous ovules; (c) a seed at the early developmental stage displaying the structure of the integument; (d–f) a female cone at middle developmental stage displaying the developing ovule with the integument thickened at the lateral side only, (d) lateral view, (e) apical view, (f) longitudinal section; (g) a female cone at late developmental stage displaying the developing seed situated in the receptacle; (h) longitudinal section of a seed at late developmental stage displaying the structure of the integument and the seed positioned in the receptacle. b, bract; i, integument; m, micropyle; o, ovule; r, ridge; s, seed. Scale bars (a–h): 1 mm. (a–b, d–e, g–h) photo by Zhi Yang; (c, f) photo by Zhi Yang and Jia‐Yi Song.

**FIGURE 3:**
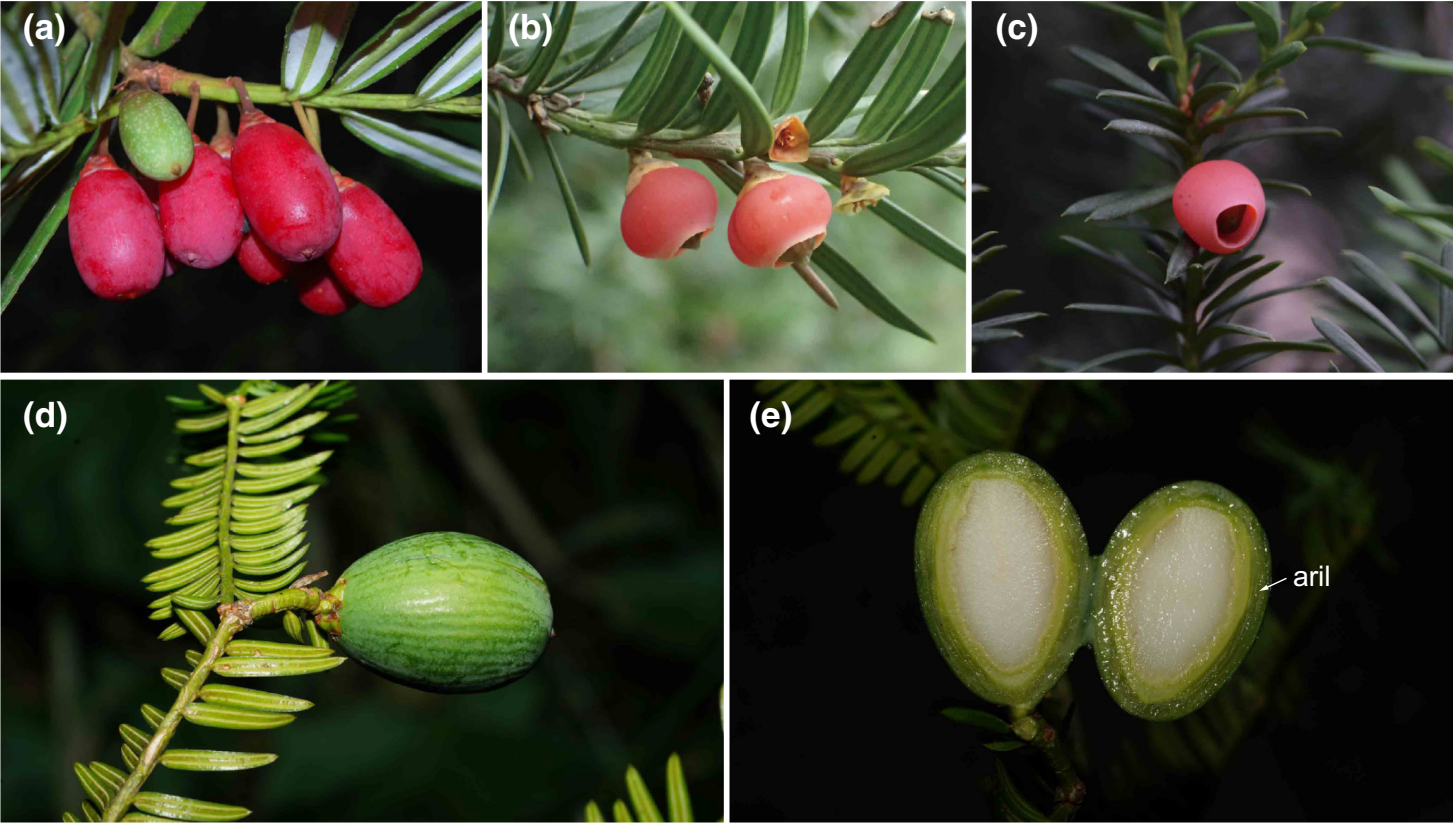
Morphology and structure of female cones of Taxaceae. (a) *Amentotaxus yunnanensis*, photo by Bing Liu; (b) *Taxus cuspidata*, photo by Yong Yang; (c) *T. chinensis*, photo by Zhi Yang; (d) lateral view of a female cone of *Torreya grandis*, photo by Yong Yang; (e) longitudinal section of a female cone of *T. grandis*, photo by Yong Yang.

The female reproductive organs of *Cephalotaxus* (Figure 2a–h) are markedly different from those of the Taxaceae (Figure 3a–e). In *Cephalotaxus*, the female organ is a pedunculate spike and consists of a thickened cone axis bearing 3–8 pairs of decussate bracts, each bract with the exception of the proximal pair subtending two axillary ovules, while a ridge or protuberance is developed between the two ovules (Figure 2a–c, Lo & Wang, 2001). The axillary two ovules represent a reduced secondary shoot (Wordsell, 1901). The female cone of *Cephalotaxus* is thus biaxial and compound (Wordsell, 1901), and close to the typical female cones of the Cupressaceae. In the Taxaceae, the ovule/seed is terminal to a twig and possesses a fleshy aril derived from the fusion of a pair of bracts (e.g., *Pseudotaxus* W.C. Cheng, Dörken et al., 2019), and thus a uniaxial structure and different from the compound female cones of other conifers.

**FIGURE 4:**
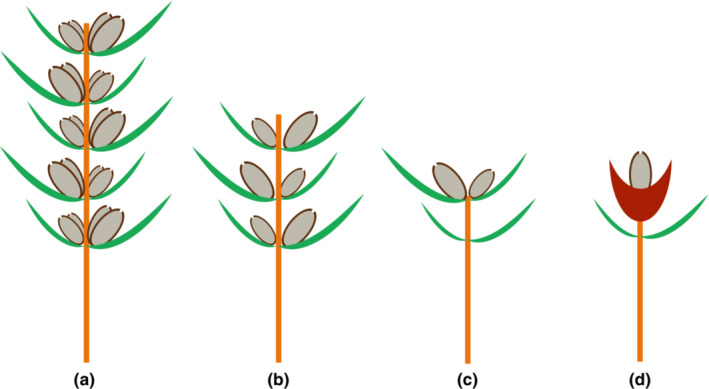
Suggested evolution of female cones of Cephalotaxaceae. (a) a diagramed female cone of Cephalotaxaceae; (b, c) hypothetical evolutionary stages of female cones toward Taxaceae; (d) a diagramed female cone of Taxaceae.

Florin (1951a, 1951b, 1954) proposed the well‐known seed scale complex evolutionary model and suggested that conifers are characterized by compound female cones possessing a cone axis with a number of seed scale complexes, the seed scale complex of conifers originated from the spike‐like secondary reproductive shoot in Cordaitales by means of evolutionary changes including shoot symmetry from radial to flattened, aggregation of fertile organs to the adaxial side and sterile foliar organs to the abaxial side, and reduction and fusion of the foliar and axial structures. In the female cones of *Cephalotaxus*, a fertile bract and its axillary two ovules constitute a unit (seed scale complex), which is decussately positioned along the cone axis (Figure 2a,b, Chen, 1999; Lo & Wang, 2001). Vascular anatomy gives some clues in this respect. In conifers, a foliar organ normally receives one vascular bundle, while an axial organ receives two vascular bundles (Chen, 1999). There are three vascular bundles entering the base of a fertile bract and its axillary two ovules, the fertile bract receiving one bundle and each ovule receiving one bundle, suggesting that the bract is foliar in nature and the two ovules represent two foliar megasporophylls of the secondary axis (Chen, 1999). The ridge/protuberance between the two ovules (Figure 2b) may be the residual apex of the secondary shoot (Chen, 1999; Lo & Wang, 2001). Stützel and Röwekamp (1999) compared the long shoot of the female reproductive organs of *Torreya* as a whole to the female cone of *Cephalotaxus* and believed that there is no fundamental difference between *Cephalotaxus* and *Torreya*. Despite this comparison, a morphological gap remains between the female cone of the Cephalotaxaceae and the terminal, uniaxial, single‐ovuled cone of the Taxaceae. During the evolutionary process, transitional morphology including the following stages may have existed: (1) the ancestral female cone evolved from multiple pairs of fertile bracts to one distal pair of fertile bracts; (2) reduction of the female cone from multiple ovules to one ovule and shifting of ovule position from axillary to terminal; (3) the pair of bracts below the seed fused to become fleshy at maturity in adaptation to bird dispersal (Figure 4).

Female reproductive organs of extant gymnosperms possess a fleshy part at maturity, for example, the sarcotesta of cycads and *Ginkgo*, the receptacle and the epimatium of the Podocarpaceae, the seed scale complex of Junipers, and the aril of the Taxaceae, the bract of the Ephedraceae, and the outer envelope of Gnetaceae (Bierhorst, 1971; Nigris et al., 2021). It is suggested that fleshy structures of gymnosperms are associated with ovule protection and seed dispersal (Nigris et al., 2021). Some people believed that the reduced fleshy female cones of conifers were derived because of common ancestry (e.g., Keng, 1975). However, molecular phylogenetic studies based on DNA sequences have consistently suggested that the fleshiness of conifers actually evolved multiple times, in Podocarpaceae, Junipers, Cephalotaxaceae, and Taxaceae, respectively (Nigris et al., 2021).

Different opinions exist on the origin of the fleshy part related to seeds of *Cephalotaxus*. Cheng and Fu (1978) suggested that the succulent part is derived from the basal cushion‐like receptacle below the ovule. If this is the case, then the fleshy aril in *Cephalotaxus* is similar to the cup‐like aril in the Taxaceae. Eckenwalder (2009) indicated “the fleshy outer layer of the seed coat intimately united with a surrounding fleshy aril that extends almost all the way to the tip.” Dörken et al. (2019) also considered the fleshy part of *Cephalotaxus* as an aril homologous to the aril of the Taxaceae. Singh (1978) and Biswas and Johri (1997), however, contended that the differentiation of the mature integument into three layers, the outer sarcotesta becoming fleshy and forming the fleshy layer in *Cephalotaxus*, distinguishes it from the aril of the Taxaceae while showing similarities to the sarcotesta of cycads and *Ginkgo* L. Li et al. (1986) stated that the seed of Cephalotaxaceae possesses no aril. We observed the structure and development of the seed coat development of *Cephalotaxus* (Figure 2a–h), and agreed with Singh (1978), Biswas and Johri (1997), and Yang et al. (2011) that the seed coat of *Cephalotaxus* at maturity is differentiated into three layers, viz. endotesta, sclerotesta, and sarcotesta; the sarcotesta is thus different from the aril in the Taxaceae in origin, the fleshy layer of the Cephalotaxaceae being part of the seed coat (developing from integument), while the aril of the Taxaceae is derived from a modification of bracts, for example, *Pseudotaxus* (Dörken et al., 2019).

Male cones are clustered in globose groups in the axil of leaves in the family Cephalotaxaceae and are thus markedly different from the simple male cones with microsporophylls inserted along the cone axis in the Taxaceae. Florin (1948) suggested that the radial perisporangiate microsporophylls are more primitive than the dorsiventral hyposporangiate microsporophylls in the Taxaceae. Morphological and developmental studies have suggested the derivation of the “simple” male cones of *Taxus* and *Torreya* from the ancestral compound cluster of male cones of the Cephalotaxaceae by means of a few evolutionary steps (Dörken et al., 2011; Mundry & Mundry, 2001; Wilde, 1975). At the beginning of this evolutionary process, the lateral cones of a compound male cone cluster in Cephalotaxaceae were reduced to a perisporangiate sporangiophore, for example, in *Taxus* (Dörken et al., 2011; Mundry & Mundry, 2001; Wilde, 1975). Then, the adaxial sporangia of the perisporangiate sporangiophore got further reduced to give rise to the hyposporangiate (dorsiventral) sporangiophore in *Torreya* (Dörken et al., 2011; Mundry & Mundry, 2001; Schulz et al., 2014; Wilde, 1975). As a result, the microsporangium‐bearing structure of the Taxaceae is not foliar but axial and equivalent to a pseudanthium (Dörken et al., 2011; Mundry & Mundry, 2001; Schulz et al., 2014). *Pseudotaxus chienii* (W.C. Cheng) W.C. Cheng occupies a transitional position in this evolutionary process (Dörken et al., 2011). According to this reduction and alteration hypothesis, the family Cephalotaxaceae can be considered as the outgroup of the Taxaceae, and as in the female reproductive organs, the male reproductive organs are also extremely reduced in the family Taxaceae. Pollen morphology also supports the Cephalotaxaceae being more primitive than the Taxaceae (Xi, 1993).

## 3 ANATOMY

The Cephalotaxaceae are similar to the Taxaceae in wood anatomy, for example, the growth rings are inconspicuous, the axial tracheids have spiral thickening, the horizontal wall of wood ray tracheids possess simple pits, the cross‐field pits being either piceoid or cupressoid, and also in the type, distribution, and arrangement of stomata, and structure of leaves (Hu, 1999). In addition, both possess transfusion tissue, a kind of tissue occurring only in gymnosperms. It lies between leaf veins and vascular bundle sheath or endodermis (Hu, 1999). Seven types of transfusion tissue have been recognized in gymnosperms: *Pinus* type, *Pseudotsuga* type, *Tsuga* type, *Cupressus* type, *Taxus* type, *Araucaria* type, and *Cycas* type (Hu, 1999). The transfusion tissue of the Cephalotaxaceae belongs to the *Taxus* type and is similar to that in the Taxaceae (Hu & Yao, 1981). However, the Cephalotaxaceae can be distinguished from the Taxaceae by the vascular anatomy of the female cones, the ovule of Cephalotaxaceae being supplied by two inverted bundles, whereas the ovule of the Taxaceae is supplied by a variable number of normally oriented bundles (Singh, 1978). Yadav et al. (2013) suggested that the presence of axial parenchyma is different in Taxaceae and Cephalotaxaceae, being absent in the former but present in the latter family. It is worth mentioning that *Cephalotaxus* is similar to *Torreya* and *Amentotaxus* in the presence of resin canals in leaves on the one hand, and similar to *Austrotaxus*, *Pseudotaxus*, and *Taxus* in lacking vascular sclereids on the other hand (Elpe et al., 2018). These characters make sense in a phylogenetic context that *Cephalotaxus* is sister to the Taxaceae.

## 4 EMBRYOLOGY

Singh (1961, 1978) suggested that embryological characters do not support Florin's opinion of the isolated position of *Cephalotaxus* among the conifers, the unwinged pollen and the absence of prothallial cells in male gametophyte are similar to those of cupressads, taxads, and taxodiads (i.e., Cupressidae). The lack of prothallial cells in the male gametophyte of *Cephalotaxus* was already noticed by Lawson (1907). Chen and Wang (1990) summarized the important similarities between Cephalotaxaceae and Taxaceae: (1) the stellate structure is present in the spermatogenous cell, (2) the nucleoloid structure is randomly distributed in both cytoplasm of pollen tube and egg, and the nucleoloids are all released into the egg together with sperms, sterile cell, tube nucleus, and starch grains in the pollen tube, (3) fusion of both nuclei is completed after mitosis of male and female gametes in the process of fertilization, (4) the proembryo has 16 free nuclei except in *Torreya*. Chen (1999) emphasized that compared with other conifer families, the Cephalotaxaceae show more similarities to the Taxaceae in embryology, and the two families should be united if only embryological characters are considered.

However, it is notable that there are a number of embryological distinctions between the two families. Pavement tissue is present in ovules of Cephalotaxaceae but absent in Taxaceae (Singh, 1978). Archegonia are long, narrow, and pointed at the chalazal end in Cephalotaxaceae but short and blunt in Taxaceae (Singh, 1978). There are 16 free nuclei in the proembryo of the Cephalotaxaceae (Li et al., 1986) but only eight free nuclei i n that of the Taxaceae (Singh, 1978). The young embryo possesses prominent cap cells in the Cephalotaxaceae, while these are absent in the Taxaceae (Singh, 1978). Chen and Wang (1990) stated that the number of free nuclei of the female gametophyte shows the most remarkable difference between the Cephalotaxaceae and Taxaceae, there being 1024 to 4096 free nuclei before wall formation in the Cephalotaxaceae but only 256 in Taxaceae. They thus concluded that the family Cephalotaxaceae is more primitive than the Taxaceae.

It is general in gymnosperms that the development of the microgametophyte finally gives rise to two sperms. The relative size of the two sperms is variable in the Cupressidae and may have evolutionary significance. The two sperms of Cupressaceae are equal in size, which is a primitive feature (Chen & Wang, 1990). In the Cephalotaxaceae, the two sperms are more or less unequal in size (Chen et al., 1987; Li et al., 1986). In the Taxaceae, the two sperms are very different in size, one is much larger than the other, and only the big sperm is functional (Chen et al., 1987). Chen and Wang (1990) argued that the evolutionary trend in sperm morphology is from equal to unequal in size in conifers, with the Cephalotaxaceae showing a transitional stage in the differentiation of the two sperms between Cupressaceae and Taxaceae. However, it should be noted that Anderson and Owens (1999) reported that the two sperms in *Taxus brevifolia* Nutt. are of equal size, a finding corroborated by Wang et al. (2008) in *T. yunnanensis* W.C. Cheng et L.K. Fu (=*T. wallichiana* Zucc.). As a result, it remains unclear whether the two sperms are of equal size or not; the evolutionary trend suggested by Chen and Wang (1990) needs to be verified with further embryological observations.

## 5 CHEMISTRY

Chemical components are very diverse in conifers. Different families of conifers possess distinct sets of chemical constituents, and taxa with closer affinities normally possess greater similarities in their chemical constituents. Many different compounds were discovered in plants of *Cephalotaxus* species, these include alkaloids, essential oil, lignans, phenylpropanoids, terpenoids, and flavonoids (Abdelkafi & Nay, 2012; Jiang et al., 2021).

It is well‐known that *Cephalotaxus* contains alkaloids and flavones (including biflavones), these chemicals possess taxonomic value. Biflavones have been found in the Cephalotaxaceae and most other conifer families excepting Pinaceae (Hao et al., 2021; Ma et al., 1990; Ma & He, 1999; Mei et al., 2006; Ren et al., 2018), supporting the separation of conifers into two clades, Pinidae (including only Pinaceae) and Cupressidae (including Araucariaceae, Podocarpaceae, Sciadopityaceae, Cupressaceae, Cephalotaxaceae, and Taxaceae).

Alkaloids constitute the second major chemical constituent in the Cephalotaxaceae. More than 30 kinds of alkaloids have been isolated and identified so far (Zhu & Zhu, 1999). These alkaloids are mainly classified into two groups, that is, cephalotaxine and homoerythrine‐type alkaloids (Chu, 1979; Hao et al., 2021; Mei et al., 2006; Zhu & Zhu, 1999). The Cephalotaxaceae are distinguished from the Taxaceae by the types of the alkaloids. The Taxaceae possess no cephalotaxine and homoerythrine‐type alkaloids but have taxine and pseudoephedrine (Chu, 1979; Zhu & Zhu, 1999), which corroborates the taxonomic separation of Cephalotaxaceae from Taxaceae.

In addition, diterpenoids were isolated from *Cephalotaxus fortunei* (Hao et al., 2021; Jiang et al., 2021; Zhang et al., 2021). They were identified as 12,16‐dihydroxyabieta‐6,8,11,13‐tetraene‐3‐one, 6α‐hydroxy sandaracopimaric acid, cephafortoid B, hainanolidol, hainanolide, hinokiol, lanceolatin D, margoclin, sugiol, and torreyayunnin (Zhang et al., 2021). This class of compounds has received much attention recently because of their remarkable antitumor effects (Jiang et al., 2021).

The plum yews are economically important not only because of the excellent timber utilized for construction and furniture (Wang & Wang, 1992) but because plants of *Cephalotaxus* contain important alkaloids, for example, cephalotaxines (Wang & Wang, 1992). The characteristic chemical components of *Cephalotaxus* including cephalotaxines were found to have important pharmacological effects and could be used to treat patients with acute and chronic diseases such as leukemia and lupus erythematosus (Mei et al., 2006; Zhang et al., 2011). To extract the effective chemicals, *Cephalotaxus* species in China were over‐exploited and many wild populations went extinct (Wang & Wang, 1992). Two species, viz. *C. lanceolata* and *C. oliveri* are included in the updated list of National Key Protected Wild Plant Species released in September of 2021 (http://www.forestry.gov.cn/main/5461/20210908/162515850572900.html). A conflict has arisen between the utilization of natural resources and the conservation of wild populations.

## 6 PHYLOGENY AND PHYLOGENOMICS

**FIGURE 5:**
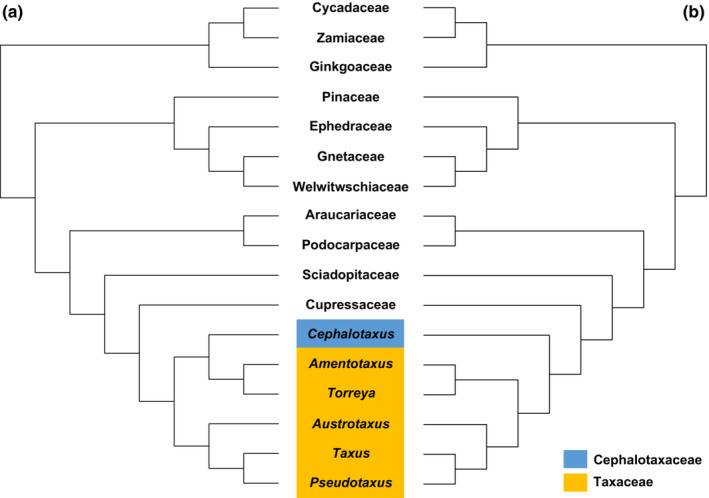
Phylogenetic trees showing relationships of Cephalotaxaceae according to previous studies. (a) Cephalotaxaceae nested within Taxaceae suggested by phylogenetic studies based on a few markers (Ghimire & Heo, 2014; Lu et al., 2014); (b) sister relationships of Cephalotaxaceae and Taxaceae supported by recent phylogenomic results (Liu et al., 2022; Stull et al., 2021).

Robust phylogenies provide a backbone for reasonable taxonomic treatments. All phylogenetic studies based on DNA sequences consistently indicated that the family Cephalotaxaceae together with the Sciadopityaceae, Cupressaceae, and Taxaceae constitute a monophyletic group, viz. Cupressidae (Chaw et al., 1995; Liu et al., 2022; Rai et al., 2008; Ran et al., 2010, 2018; Stefanovic et al., 1998; Stull et al., 2021; Wang & Shu, 2000). Within the clade of the Cupressidae, the family Sciadopityaceae is the most primitive lineage, the Cupressaceae are sister to a subclade including the Cephalotaxaceae and Taxaceae. However, there are different opinions on whether Cephalotaxaceae should be incorporated into the Taxaceae. Some studies suggest that the family Cephalotaxaceae is sister to Taxaceae, whereas others suggest that the plum yew family is nested within the Taxaceae, whereas others imply that the plum yew family is sister to the Taxaceae (Figure 5).

Cladistic analyses based on 28 morphological and anatomical characters suggested that the Cephalotaxaceae should be incorporated into the Taxaceae (Ghimire & Heo, 2014). A phylogeny‐based chloroplast *trn*L‐*trn*F region indicated that *Cephalotaxus* is nested within the Taxaceae and sister to a subclade including *Amentotaxus* and *Torreya* (Hao et al., 2009). Lu et al. (2014) reconstructed a phylogeny of gymnosperms using two single‐copy nuclear genes including *LFY* and *NLY* and found that the Cephalotaxaceae are nested within the Taxaceae. A phylogeny based on RAD‐seq suggested that *Cephalotaxus* is nested within the Taxaceae (Majeed et al., 2019).

Wang and Shu (2000) established a phylogeny based on the chloroplast *mat*K and obtained a phylogenetic tree showing that the Cephalotaxaceae and Taxaceae are reciprocally monophyletic. Cheng et al. (2000) conducted a phylogenetic study using chloroplast *mat*K and nrITS and concluded that the family Cephalotaxaceae is sister to the Taxaceae. Elpe et al. (2018) conducted a phylogenetic study using nuclear ITS and *PHYP*, and plastid *mat*K and *rbc*L, and concluded that *Cephalotaxus* is not nested within the Taxaceae but is sister to it. Both plastome phylogenomics (Ji et al., 2021) and Phylogenomics using a large number of single‐copy nuclear genes (Liu et al., 2022; Ran et al., 2018; Stull et al., 2021) have indicated that the family Cephalotaxaceae is sister to but not nested within the Taxaceae.

## 7 TAXONOMY

Many different taxonomic treatments of *Cephalotaxus* have been proposed by previous researchers. Saxaton (1913) classified the conifers with atypical female cones in the family Taxaceae, which he further divided into three tribes, that is, Cephalotaxeae (*Cephalotaxus*), Taxeae (*Torreya* Arn. and *Taxus* L.), and Podocarpeae (including *Phyllocladus* Rich. ex Mirb.); he considered the Cephalotaxeae as the most primitive group in the family Taxaceae, thereby disagreeing with early classifications, which included *Ginkgo* in the family Taxaceae and considered the reduced ovulate organs of taxads and *Ginkgo* were the result of convergent evolution. Saxton's idea was generally accepted by Keng (1975), who classified the conifers with atypical female cones in the suborder Taxineae, which included four families, that is, Phyllocladaceae, Taxaceae, Podocarpaceae, and Cephalotaxaceae. Sahni (1920) emphasized the similarities of seeds in the Taxaceae and Paleozoic Cordaitales and suggested classifying *Cephalotaxus* and genera of the Taxaceae in a separate order Taxales, and believed that the reduced female organs of taxads originated from the Cordaitales. Pilger (1926) laid the foundation of the modern taxonomy of the gymnosperms. He included *Amentotaxus* Pilg. and *Cephalotaxus* in the family Cephalotaxaceae, a treatment adopted by Page (1990), while many others treated them as two separate families and included *Amentotaxus* in the Taxaceae (e.g., Biswas & Johri, 1997; Cheng & Fu, 1978). Takhtajan (1953) classified conifers in the order Coniferales of the subclass Stachyosperminae and treated the Cephalotaxaceae and Taxaceae as two separate families. Florin (1948, 1954) largely agreed with Sahni (1920) regarding the archaic nature of taxad seeds, but he preferred to separate *Cephalotaxus* from the Taxaceae with the latter in a separate order (or class) Taxales. Pilger and Melchior (1954) classified conifers into two classes, viz. Coniferopsida and Taxopsida, and included Cephalotaxaceae in the class Coniferopsida with only Taxaceae in the class Taxopsida. Sporne (1971), Stewart (1983), and Biswas and Johri (1997) made a similar treatment, both classified Cephalotaxaceae in the Coniferales and retained only Taxaceae in the Taxales. Cheng and Fu (1978) treated *Cephalotaxus* as a separate order Cephalotaxales intermediate between the Podocarpales and the Taxales in the class Coniferopsida. Christenhusz et al. (2011) adopted earlier phylogenetic results based on DNA fragments and incorporated *Cephalotaxus* into the Taxacae. Ghimire and Heo (2014) and Ghimire et al. (2018) conducted cladistic analyses based on morphological/anatomical characters, which led them to incorporate *Cephalotaxus* in the Taxaceae. Yang et al. (2022), however, considered both recent phylogenomic results and phenetic characters and classified *Cephalotaxus* as a separate family, which represents the latest and most convincing solution to the taxonomy of gymnosperms.

## 8 CONCLUSIONS

It is concluded here that the Cephalotaxaceae should be treated as a separate family from the Taxaceae in the order Cupressales because recent phylogenomic studies have consistently shown the two families belong to a clade consisting of Sciadopityaceae, Cupressaceae, Cephalotaxaceae, and Taxaceae. Cephalotaxaceae and Taxaceae are reciprocally sister to one another and can be distinguished by the morphology of the female cones, anatomy of leaves and wood, embryology, and chemistry. The Cephalotaxaceae show transitional characters between the Cupressaceae and the Taxaceae in the Cupressidae. The female cone of Cephalotaxaceae displays certain similarities to Cupressaceae but can produce the extremely reduced female organs of Taxaceae by means of further reduction and alteration. The morphological gap between Cephalotaxaceae and Taxaceae may be represented by transitional macrofossils from the early Mesozoic (e.g., Triassic to Jurassic).

## AUTHOR CONTRIBUTIONS

**Yong Yang:** Conceptualization (lead); funding acquisition (lead); investigation (lead); project administration (lead); supervision (lead); writing – original draft (lead); writing – review and editing (lead). **Zhi Yang:** Data curation (lead); formal analysis (lead); investigation (equal); methodology (lead); software (lead); visualization (lead). **David Kay Ferguson:** Investigation (supporting); writing – review and editing (supporting). **Jia‐Yi Shong:** Investigation (supporting).

## CONFLICT OF INTEREST STATEMENT

The authors declare that there is no conflict of interest.

## Data Availability

All data used in the study are included in this paper.
